# Supplementary material for: Characterization of Stealth Liposome-Based Nanoparticles Encapsulating the ACAT1/SOAT1 Inhibitor F26: Efficacy and Toxicity Studies In Vitro and in Wild-Type Mice
Source: Int J Mol Sci. 2024 Aug 23;25(17):9151. doi: 10.3390/ijms25179151 (PMC11394700; doi:10.3390/ijms25179151)
Supplement: Supplementary file 1 [file ijms-25-09151-s001.zip › ijms-3150821-supplementary.pdf]

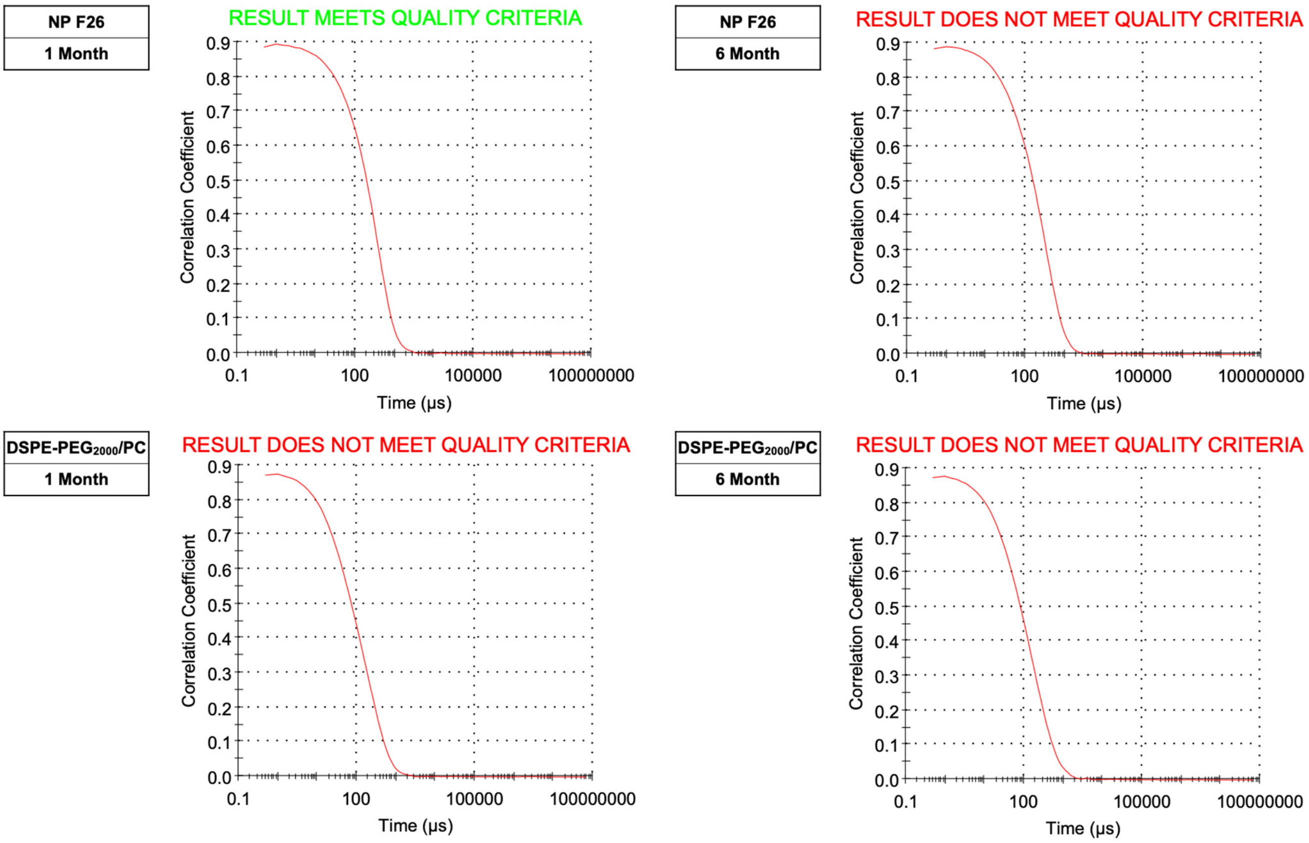

Figure S1. Size quality reports of NP F26 and DSPE-PEG$_{2000}$/PC nanoparticles. Results were obtained using the Zetasizer Nano ZS software. Correlograms exhibited a smooth exponential decay to a flat baseline, where the slope of decay reflects the polydispersity of the particle size distribution. NP F26 at 1 month met the quality criteria set by the manufacturer's software. NP F26 at 6 months displayed increased polydispersity, failing to meet the quality criteria. In contrast, DSPE-PEG$_{2000}$/PC nanoparticles, at both 1 month and 6 months, were highly dispersed compared to NP F26, as detailed by their size distributions in Figure 1D.
